# Supplementary material for: Mathematical Approach to Estimating the Main Epidemiological Parameters of African Swine Fever in Wild Boar
Source: Vaccines (Basel). 2020 Sep 12;8(3):521. doi: 10.3390/vaccines8030521 (PMC7563513; doi:10.3390/vaccines8030521)
Supplement: Supplementary file 1 [file vaccines-08-00521-s001.pdf]

vaccines

MDPI

Supplementary Materials: The following are available online at www.mdpi.com/xxx/s1,

Table S1: Data on wild boar that were African swine fever virus (ASFV) tested for both virology and serology during the last three hunting season (from November to January), presented as contingency table with number of wild boar (percentage) for each category. Data about animals not tested for both serology and virology were excluded.

		Virus positive	Virus negative	Total
Hunting season 2011–2012	**Seropositive**	0 (0)	0 (0)	0
	**Seronegative**	1 (1.4)	70 (98.6)	71
	**Total**	1	70	71
Hunting season 2012–2013	**Seropositive**	0 (0)	4 (3.2)	4
	**Seronegative**	1 (0.8)	120 (96)	121
	**Total**	1	124	125
Hunting season 2013–2014	**Seropositive**	3 (0.6)	6 (1.2)	9
	**Seronegative**	14 (2.9)	454 (95.2)	468
	**Total**	17	460	477
Hunting season 2014–2015	**Seropositive**	2 (0.6)	13 (4.0)	14
	**Seronegative**	1 (0.3)	307 (95.0)	309
	**Total**	3	320	323
Hunting season 2015–2016	**Seropositive**	3 (0.4)	23 (3.9)	26
	**Seronegative**	0 (0)	583 (95.7)	583
	**Total**	3	606	609
Hunting season 2016–2017	**Seropositive**	0 (0)	29 (2.9)	29
	**Seronegative**	0 (0)	973 (97.1)	973
	**Total**	0 (0)	1002	1002
Hunting season 2017–2018	**Seropositive**	0 (0)	12 (1.1)	12
	**Seronegative**	0 (0)	1142 (98.9)	1142
	**Total**	0 (0)	1154	1154
Hunting season 2018–2019	**Seropositive**	0 (0)	8 (0.6)	8
	**Seronegative**	0 (0)	1248 (99.3)	1248
	**Total**	0 (0)	1256	1256
Hunting season 2019–2020	**Seropositive**	0 (0)	5 (0.4)	5
	**Seronegative**	0 (0)	1303 (99.6)	1303
	**Total**	0 (0)	1308	1308

Table S2. Summarize of baseline database created for the passive surveillance in Anglona-Gallura area, including the year (from 2011 to 2020), number of found dead wild boar, number of those tested for African swine fever virus (ASFV) presence or antibody presence, and the number of animals virus positive or seropositive. Data are presented as number and percentage.

Year	Wild boar found dead	Wild boar virologically[1] tested (n, %)	Virus positive	Wild boar serologically[2] tested (n, %)	Seroprositive[3]
**2011**	10	3 (30.0)	0 (0)	7 (70.0)	0 (0)
**2012**	10	6 (60.0)	0 (0)	6 (60.0)	0 (0)
**2013**	17	7 (41.2)	0 (0)	8 (47.0)	0 (0)
**2014**	29	23 (79.3)	0 (0)	9 (31.0)	0 (0)
**2015**	21	14 (66.7)	0 (0)	15 (71.4)	0 (0)
**2016**	24	19 (79.1)	0 (0)	9 (37.5)	0 (0)
**2017**	24	19 (79.1)	0 (0)	15 (62.5)	0 (0)

**2018**	17	17 (100)	0 (0)	8 (47.0)	0 (0)
**2019**	76	75 (98.7)	0 (0)	25 (32.9)	0 (0)
**2020**	40	35 (87.5)	0 (0)	18 (45.0)	0 (0)
**Total**	**268**	**218 (81.3)**	**0 (0)**	**120 (44.8)**	**0 (0)**

[1] Virus presence was assessed by Real Time PCR, Quantitative PCR or Malmquist test. [2] Wild boar serologically tested are those tested with at least screening ELISA test (Ingezim PPA Compac ®, Ingenasa, Madrid, Spain), and eventually confirmed (if positive) with immunoblotting (IB) test, in accordance to the Manual of diagnostic test and vaccines from terrestrial animals. [3] Serum samples were considered positive when they scored positive in both the screening (ELISA) and the confirmatory (IB) tests.
